# A Rare Cause of Jaundice Following Cholecystectomy: Compression by the Silicon Drain

**DOI:** 10.4021/gr504e

**Published:** 2012-11-20

**Authors:** Fevzi Cengiz, Savas Yakan, Mehmet Akif Ustuner, Abdullah Senlikci, Enver Ilhan

**Affiliations:** a Izmir Bozyaka Educational and Research Hospital, General Surgery Clinic, Turkey

**Keywords:** Drain management, Obstructive jaundice, Extrahepatic bile duct obstruction

## Abstract

Jaundice that develops following laparoscopic cholecystectomy is a troublesome experience for the surgeon which requires invasive management after a challenging diagnosis period. Jaundice is. We aimed to present our experience with a rare complication of jaundice in a patient that occurred due to the compression of an isolated drain without choledoc canal injury. A 63-year-old female patient underwent laparoscopic cholecystectomy due to symptomatic gallstone. The patient developed post-operative jaundice which was detected by upper abdominal magnetic resonance (MR) and magnetic resonance cholangiopancreatography (MRCP) to result from compression by the silicon drain on main hepatic canal. The patient was discharged upon removal of the silicon drain with recovery in biochemical and radiological parameters. To the best of our knowledge, our study is the first to report jaundice developing due to extrahepatic bile duct obstruction caused by isolated drain compression. Although this rare complication can be diagnosed by radiological workup and managed by simple surgical intervention, we believe that it requires consideration among other possible complications during laparoscopic cholecystectomy.

## Introduction

Obstructive jaundice following laparoscopic cholecystectomy is a troublesome experience which requires invasive management after a challenging diagnosis period. Bile duct injury is a common etiology in cases lacking choledocholithiasis [1]. Obstruction rarely is due to an unknown cause resulting in self-limiting inflammation [2]. The objective of this study is to present a case who developed self-limiting obstruction due to the silicon drain compression which has not been reported in the literature following laparoscopic cholecystectomy.

## Case Report

**Figure 1:**
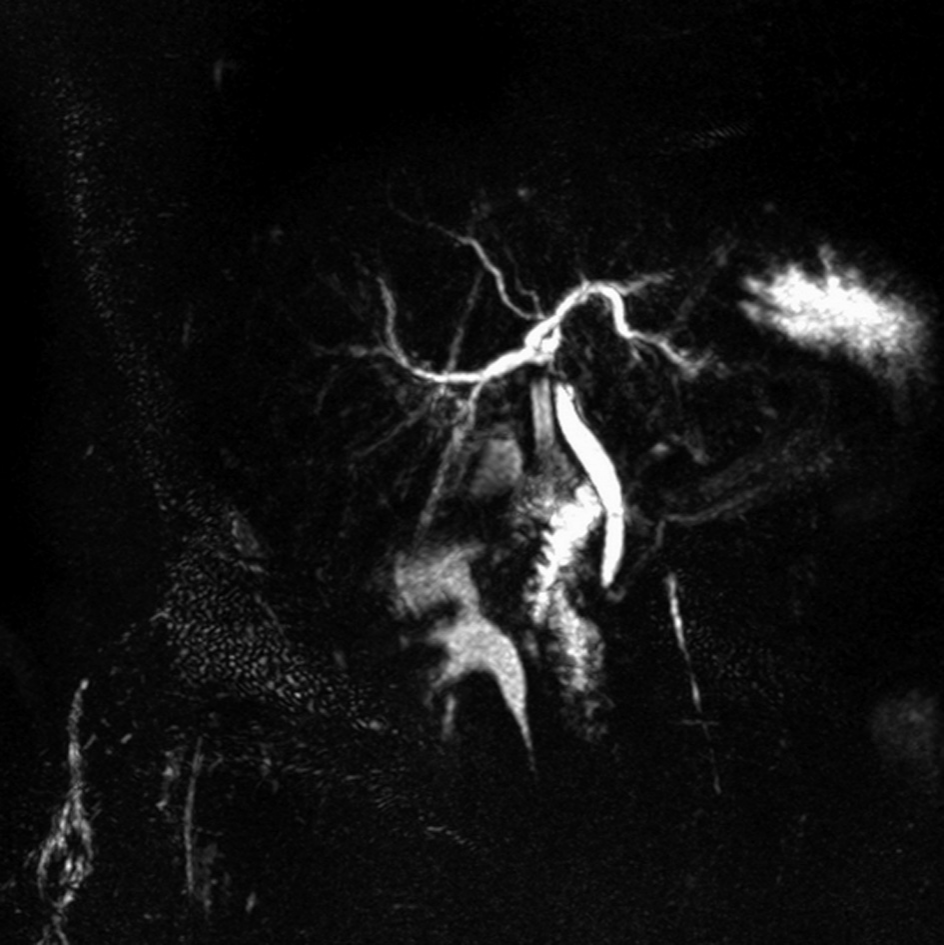
MRCP scan showing obstruction on the proximal portion of the main hepatic canal suggesting a compression due to silicon drain.

**Figure 2:**
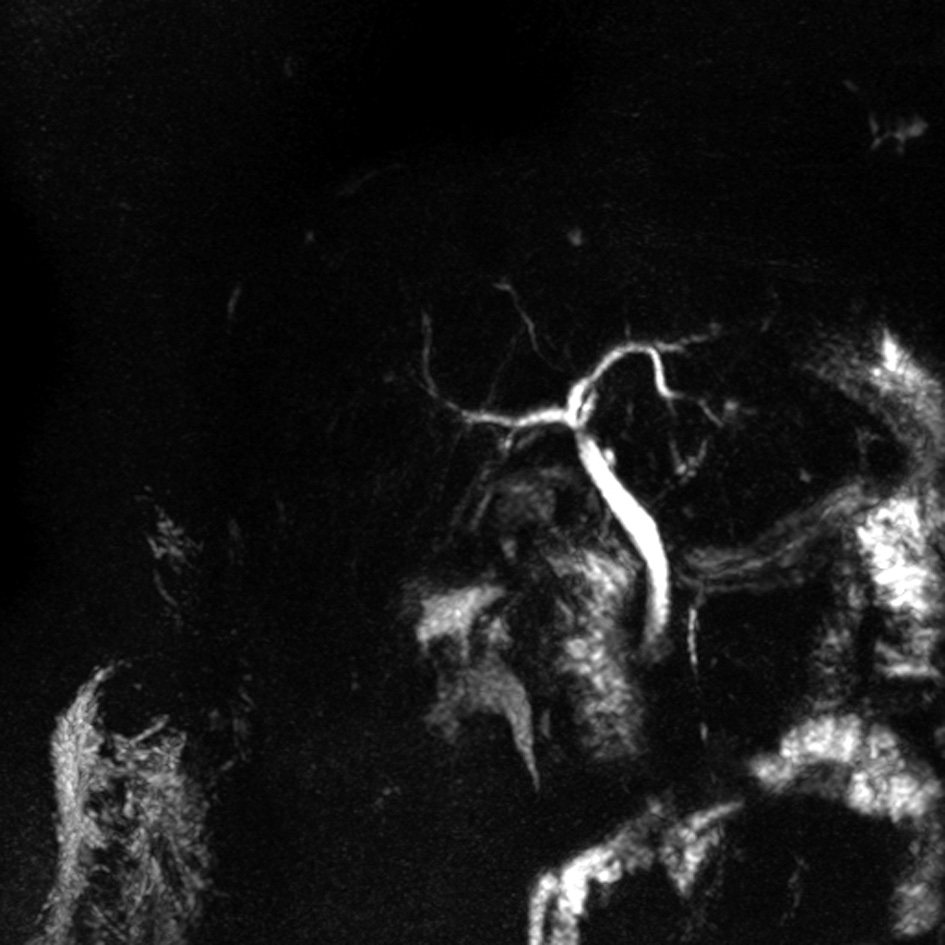
Following removal of the silicon drain and repeat MRCP revealed disappearance of the obstruction.

A 63 year-old female patient underwent laparoscopic cholecystectomy due to symptomatic gallstone. The patient’s pre-operative biochemical workup was normal and no hepatic, choledoc or cystic canal abnormality was detected intra-operatively. The operation was performed without any complication. The patient’s total bilirubin, direct bilirubin, GGT and AST values were raised to 3.81 mg/dL, 2.94 mg/dL, 106 U/L and 193 U/L one day after the operation. She did not define abdominal pain and her vital signs were stable. Upper abdominal MR and MRCP revealed obstruction on the proximal portion of the main hepatic canal suggesting a compression due to the silicon drain placed during the operation (Fig. 1). Following immediate removal of the silicon drain total bilirubin value were reduced to 1.22 mg/dL on the same day and repeat MRCP that was performed the next day revealed disappearance of the obstruction (Fig. 2). The patient was discharged without complication on the post-operative third day with normal biochemical and radiological workup and no complication was detected on her follow-up.

## Discussion

To the best of our knowledge, our study is the first to report jaundice developing due to extrahepatic bile duct obstruction caused by isolated drain compression. Bilomas are the most common and alarming complication following laparoscopic cholecystectomy. Biloma is reflected clinically by upper right abdominal pain, nausea, vomiting and fever. However, no clinical symptom or sign was detected in our patient. The hyperbilirubinemia and laboratory findings of deteriorated liver function following laparoscopic cholecystectomy are suggestive of bile duct obstruction by a gallstone or a surgical failure [3]. Extrahepatic bile ducts must be visualized by radiological imaging in cases with similar symptoms and laboratory findings. Although the etiology of biliary obstruction can be investigated by abdominal ultrasound, computerized tomography (CT) or MR imaging, MRCP is considered as the most appropriate non-invasive diagnostic method which does no emit radiation [4]. Bile duct injuries are troublesome complications that could be managed by gallstone removal by extraction or stent placement using Endoscopic Retrograde Cholangiopancreatography (ERCP). In addition, they may require urgent reconstruction surgery due to complications such as stricture, recurrent cholangitis and secondary biliary cirrhosis. Therefore, in order to accelerate the diagnostic process, we planned urgent MRCP in our patient. Partial biliary obstruction may resolve spontaneously within days or weeks [2] with mostly unknown etiology that is attributed to local inflammation or ductal spasm. However, the etiology in our patient did not remain speculative and we detected silicon drain compression by performing urgent MRCP. Routine drain placement following elective laparoscopic cholecystectomy has been reported to enhance operative time, post-operative pain and hospital stay and, therefore, suggested as irrational and unnecessary in the literature [5]. In addition to its helplessness with regard to preventing other complications, silicon drain placement may cause inflammation in the subhepatic space leading to local and temporary partial biliary obstruction that could not be generally diagnosed.

In conclusion, to the best of our knowledge, jaundice developing due to extrahepatic bile duct obstruction caused by isolated drain compression has been reported for the first time in the literature by our study. Although this rare complication can be diagnosed by radiological workup and managed by simple surgical intervention, we believe that it requires consideration among other possible complications during laparoscopic cholecystectomy.
